# Multicolor imaging of calcium-binding proteins in human kidney stones for elucidating the effects of proteins on crystal growth

**DOI:** 10.1038/s41598-021-95782-1

**Published:** 2021-08-26

**Authors:** Yutaro Tanaka, Mihoko Maruyama, Atsushi Okada, Yoshihiro Furukawa, Koichi Momma, Yuki Sugiura, Rie Tajiri, Koichi P. Sawada, Shunichi Tanaka, Kazufumi Takano, Kazumi Taguchi, Shuzo Hamamoto, Ryosuke Ando, Katsuo Tsukamoto, Masashi Yoshimura, Yusuke Mori, Takahiro Yasui

**Affiliations:** 1 Department of Nephro-urology, Graduate School of Medical Sciences, Nagoya City University, 1-Kawasumi, Mizuho-cho, Mizuho-Ku, Nagoya, 467-8601 Japan; 2 Institute for Advanced Co-Creation Studies, Osaka University, 2-1, Yamadaoka, Suita, 565-0871 Japan; 3 Graduate School of Engineering, Osaka University, 2-1, Yamadaoka, Suita, 565-0871 Japan; 4 Graduate School of Life and Environmental Sciences, Kyoto Prefectural University, 1-5, Hangi-cho, Shimogamo, Sakyo-ku, Kyoto, Kyoto 606-8522 Japan; 5 Department of Earth Science, Tohoku University, 6-3 Aza-Aoba, Aramaki, Aoba-ku, Sendai 980-8578 Japan; 6 National Museum of Nature and Science, 4-1-1 Amakubo, Tsukuba, 305-0005 Japan; 7 Health and Medical Research Institute, National Institute of Advanced Industrial Science and Technology (AIST), 2217-14, Hayashi-cho, Takamatsu, Kagawa 761-0395 Japan; 8 Tajiri Thin Section Laboratory, 3-1-11 Sannose, Higashiosaka, Osaka 577-0849 Japan; 9 Institute of Laser Engineering, Osaka University, 2-6, Yamadaoka, Suita City, Osaka 565-0871 Japan

**Keywords:** Biological techniques, Urology

## Abstract

The pathogenesis of kidney stone formation includes multi-step processes involving complex interactions between mineral components and protein matrix. Calcium-binding proteins in kidney stones have great influences on the stone formation. The spatial distributions of these proteins in kidney stones are essential for evaluating the in vivo effects of proteins on the stone formation, although the actual distribution of these proteins is still unclear. We reveal micro-scale distributions of three different proteins, namely osteopontin (OPN), renal prothrombin fragment 1 (RPTF-1), and calgranulin A (Cal-A), in human kidney stones retaining original mineral phases and textures: calcium oxalate monohydrate (COM) and calcium oxalate dihydrate (COD). OPN and RPTF-1 were distributed inside of both COM and COD crystals, whereas Cal-A was distributed outside of crystals. OPN and RPTF-1 showed homogeneous distributions in COM crystals with mosaic texture, and periodically distributions parallel to specific crystal faces in COD crystals. The unique distributions of these proteins enable us to interpret the different in vivo effects of each protein on CaOx crystal growth based on their physico-chemical properties and the complex physical environment changes of each protein. This method will further allow us to elucidate in vivo effects of different proteins on kidney stone formation.

## Introduction

Kidney stone disease is a common disorder, affecting 1.7–14.8% of the population at least once in the lifetime^1,2^. In up to 50% of cases, this disease recurs within 5 years of the first episode. Despite the importance of this health issue, preventive therapy for stone formation is unavailable. Understanding the pathogenesis of kidney stone formation is essential to reduce the occurrence and recurrence of kidney stone disease^3^. Approximately 80% of kidney stones are calcium oxalate (CaOx) stones^4,5^. CaOx stones consisted of ~ 90% of mineral phase, i.e., CaOx which is further categorized into calcium oxalate monohydrate [Ca(C_2_O_4_)·H_2_O](COM) and calcium oxalate dihydrate [Ca(C_2_O_4_)·2H_2_O](COD), and a relatively small fraction of organic matter, which has been regarded as protein matrix^6^. The pathogenesis of kidney stone formation includes multi-step processes involving complex interactions between mineral components and protein matrix^7,8^.

More than 100 species of proteins have been identified in kidney stones^9–11^. Among them, several proteins, particularly calcium-binding proteins, are known to play essential roles for CaOx stone formation processes^12–16^. The specific effects of these proteins have been investigated intensively in many steps of the stone formation, including crystal nucleation, crystal growth, crystal aggregation, and crystal adhesion, with numerous in vitro crystallization studies^17–19^. In vitro studies are useful to evaluate the effects of specific proteins on the specific steps in the stone formation. However, in the real stone formation environments, numbers of proteins work simultaneously, and the urine composition in the concentrations of proteins, calcium ion, and oxalate fluctuates. This concern motivated us to investigate real human kidney stones to find the real effects of proteins on crystal growth.

In most previous studies, the identification of the proteins in kidney stones has been conducted with mass spectroscopy after crushing and extraction^9–11^. Thus, information on the spatial distribution of proteins in the stone is lost. In very limited investigations, protein identification in kidney stones has been conducted with sliced sections of kidney stones in which CaOx crystals are completely removed by decalcification^20,21^. This approach is useful for finding the distribution of a protein in kidney stones that potentially helps to understand specific protein effects on stone formation. However, a huge amount of information on the stone formation, recorded in kidney stone crystals, is lost in this method. The importance of mineral information of kidney stones that can be acquired by the identifications of crystal phases and the crystal texture classifications conducted using slice sections and polished thin sections of kidney stones with optical microscopy has been shown by more than 70 years of previous studies^22,23^.

The absence of the analysis to evaluate protein distributions with pristine CaOx crystals in kidney stones has been a considerable obstacle for understanding the stone formation. Coordinated evaluation of protein distributions and crystal phases/morphologies can provide significant information on the history of the stone formation. Multiple immunofluorescence staining (multi-IF staining) has been used to show the distributions of two or more proteins in many types of soft tissues of biological samples^24^. Application of the technique to bone tissues, which are composed of porous calcium phosphate crystals, also provided significant insights into dynamic regulation of bone mineral homeostasis^25^. However, this has not been used to investigate kidney stones composed of dense and hard crystals, although single immunofluorescence staining has been used to show the distribution of a specific protein in decalcified kidney stone that does not retain mineral information^20,21^. This study investigated the conditions that enable the multi-IF staining of kidney stone samples retaining the original mineral information. We investigated the distribution of three different proteins, osteopontin (OPN), renal prothrombin fragment 1 (RPTF-1), and calgranulin A (Cal-A), in thin sections of CaOx stones. These proteins are common in most CaOx stones and are known as calcium-binding proteins that potentially affect the CaOx stone formation^26–28^. To the best of our knowledge, this is the first study in which multiple matrix proteins are co-visualized in a kidney stone. We further interpret the different i*n vivo* effects of each protein on CaOx crystal growth based on their distributions, physico-chemical properties, and the complex physical environment changes of each protein during CaOx stone formation.

## Results

**Figure 1:**
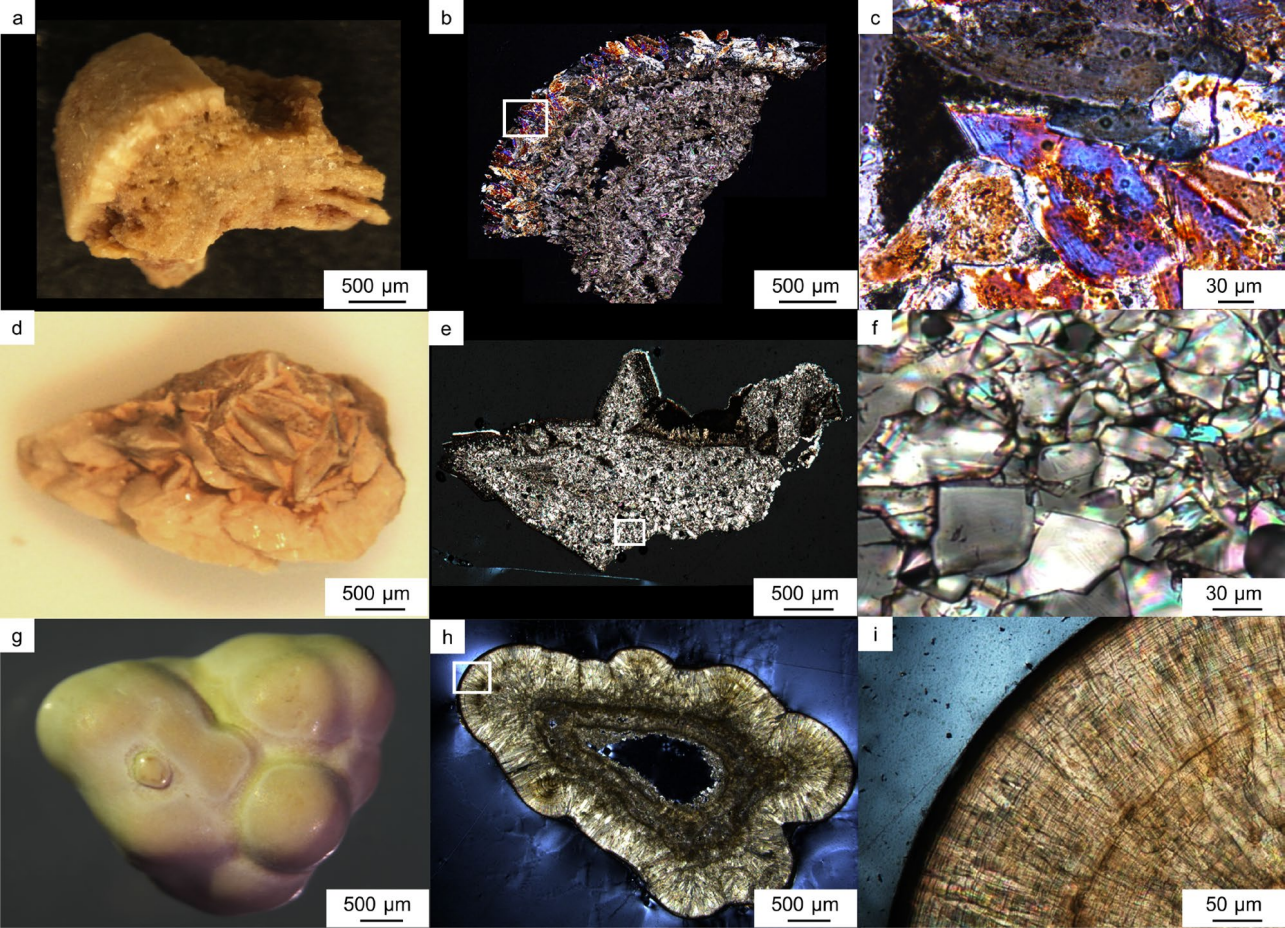
Kidney stone samples analyzed in the present study. (**a**) Sample 1. (**b**) Polarized microscopy image of the thin section of sample 1. (**c**) Enlarged image of the white box area in (**b**). (**d**) Sample 2. (**e**) Polarized microscopy image of the thin section of sample 2. (**f**) Enlarged image of the white box area in (**e**). (**g**) Sample 3. (**h**) Polarized microscopy image of the thin section of sample 3. (**i**) Enlarged image of the white box area in (**h**).

Based on microscopic observation coupled with FT-IR analysis, domains of the kidney stone samples were categorized into three types of textures that are consistent with that reported in Schubert and Brien^23^: irregular texture composed of euhedral COD crystals (Type 1, referred to as euhedral COD aggregate; Fig. 1c), mosaic texture composed of irregular oriented COM crystals (Type 2, referred to as mosaic COM; Fig. 1f), and concentrically laminated COM crystals (Type 3, referred to as concentric COM; Fig. 1i). Most of the observed CaOx stone samples consisted of these three textures (Table 1).

**Table 1:** Summary of the patient demographic, maximum size of the stone, ratio of the mineral phase.

Sample number	Age	Sex	Maximum size (mm)	CaOx (%)	Apatite (%)	Crystal texture od CaOx		
						Type 1	Type 2	Type 3
1	73	M	9	97	3	Yes	Yes	No
2	49	M	6	98	–	No	Yes	Yes
3	68	F	7	98	–	No	Yes	Yes
4	54	F	5	98	–	No	No	Yes
5	64	M	8	98	–	Yes	Yes	Yes
6	71	M	5	97	3	Yes	Yes	Yes
7	44	F	2	92	8	No	Yes	Yes
8	78	F	5	89	11	Yes	Yes	No
9	77	F	5	98	–	Yes	Yes	No
10	64	M	5	91	9	Yes	Yes	No
11	77	M	5	93	7	No	Yes	No
12	90	M	3	98	–	No	No	Yes
13	59	M	4	98	–	No	Yes	Yes
14	63	M	5	98	–	No	No	Yes
15	37	M	6	98	–	Yes	Yes	Yes

CaOx calcium oxalate, Type1: idiomorphic COD, Type2: mosaic texture composed of irregular oriented COM, and Type3: concentrically laminated COM.

Multi-IF staining protocol used for biological samples was applied to the analysis of thin sections of kidney stone samples prepared by a typical geological method. For the application, the etching condition of the thin section before the staining was adjusted and it was found that the visualization was only successful when the polished thin section was slightly etched (with a pH 6.0 citrate solution for 1 min) before the typical staining process. The multi-IF staining enabled the co-visualization of three proteins in different colors (OPN: Green, RPTF-1: Blue and, Cal-A: Red) in the three different stone textures.

**Figure 2:**
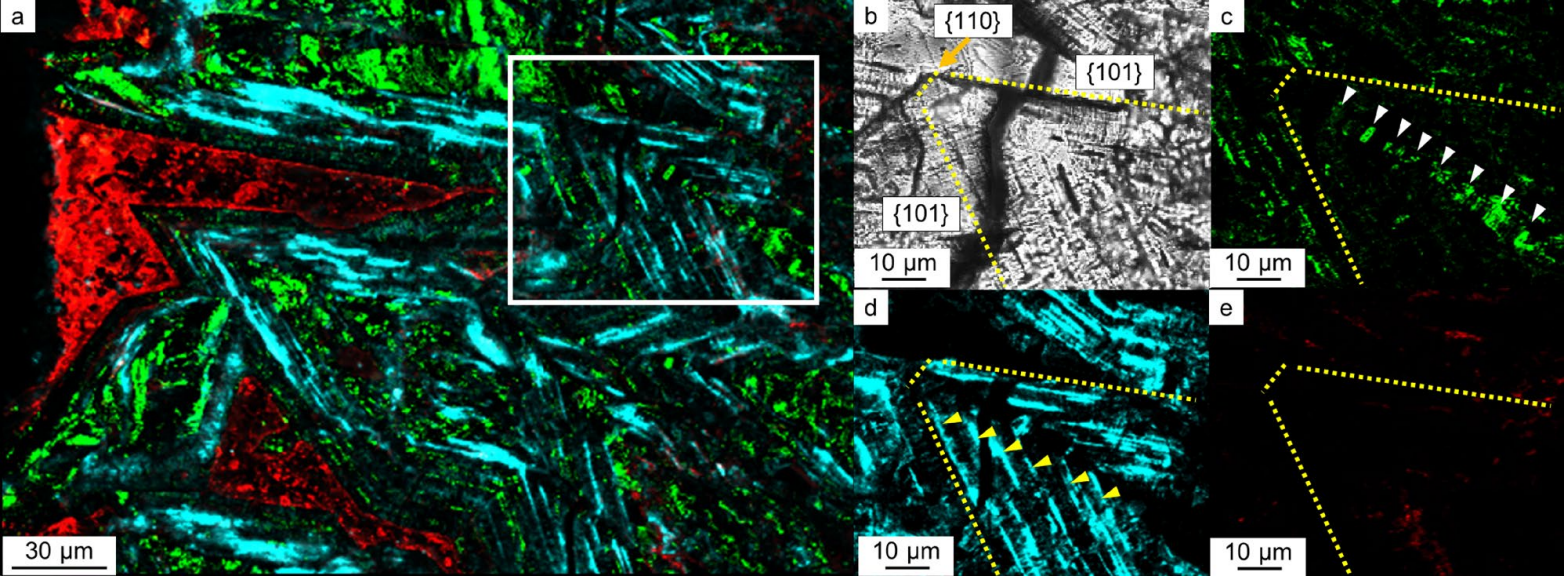
Protein distributions in euhedral COD aggregates. (**a**) Multi-IF image. Enlarged images of the white box area are shown in (**b**)–(**e**). (**b**) Optical image. Crystal faces are shown with yellow dotted lines and those indexes. (**c**) IF image of OPN. Crystal faces and laminar layers are shown with yellow dotted lines and white arrows, respectively. (**d**) IF image of RPTF-1. Crystal faces and laminar layers are shown with yellow dotted lines and yellow arrows, respectively. (**e**) IF image of Cal-A. Crystal faces and laminar layers are shown with yellow dotted lines.

We found that each protein showed a characteristic distribution pattern depending on its location in COM and COD. The euhedral COD aggregates were found in 7 samples out of 15 samples (Table 1). The euhedral COD aggregates were present predominantly on the periphery of CaOx kidney stones (Fig. 1a–c). These COD crystals are known to have a tetragonal bipyramid shape composed of {101} faces^29^. Many of the COD bipyramids also have {110} face on the tip of both pyramids (Fig. 2b and Supplementary Fig. S1). A multi-IF staining image of the COD crystals is shown in Fig. 2a, and OPN periodically presents on {110} face of COD, as shown by white arrows in Fig. 2c. This face is the same face of the bipyramid tip that does not appear in the typical COD in vitro crystal growth^29^. RPTF-1 appeared as parallel layers along the {101} face, with µm-scale intervals, as shown by the yellow arrow in Fig. 2d. This crystal face is characteristic of the typical COD growth^29^. Cal-A was present outside of the COD crystals, showing no preferential adsorption on specific faces (Fig. 2e). The same distribution patterns were seen in other stone samples (Supplementary Fig. S2).

**Figure 3:**
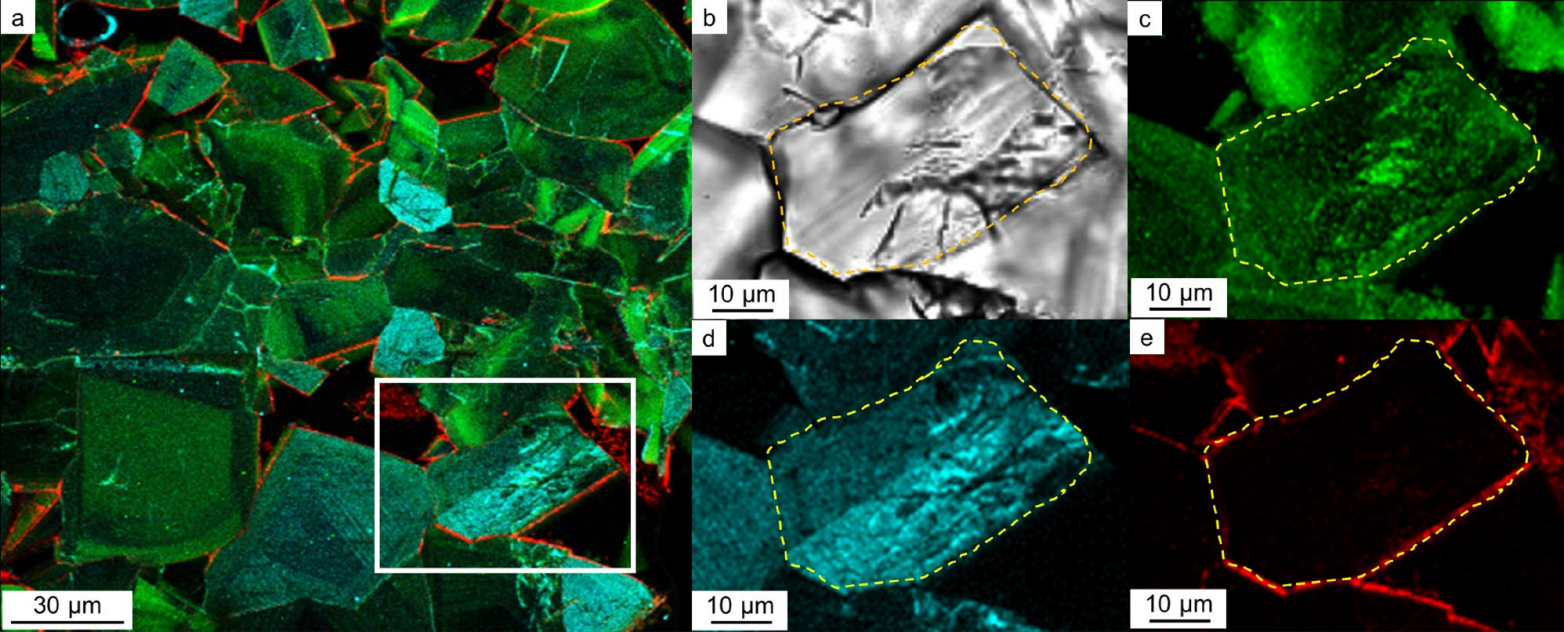
Protein distributions in a mosaic COM. (**a**) Multi-IF image. Enlarged images of the white box area are shown in (**b**)–(**e**). (**b**) Optical image. A grain boundary is shown with a yellow dashed line. (**c**) IF image of OPN. A grain boundary is shown with a yellow dashed line. (**d**) IF image of RPTF-1. A grain boundary is shown with a yellow dashed line. (**e**) IF image. A grain boundary is shown with a yellow dashed line.

**Figure 4:**
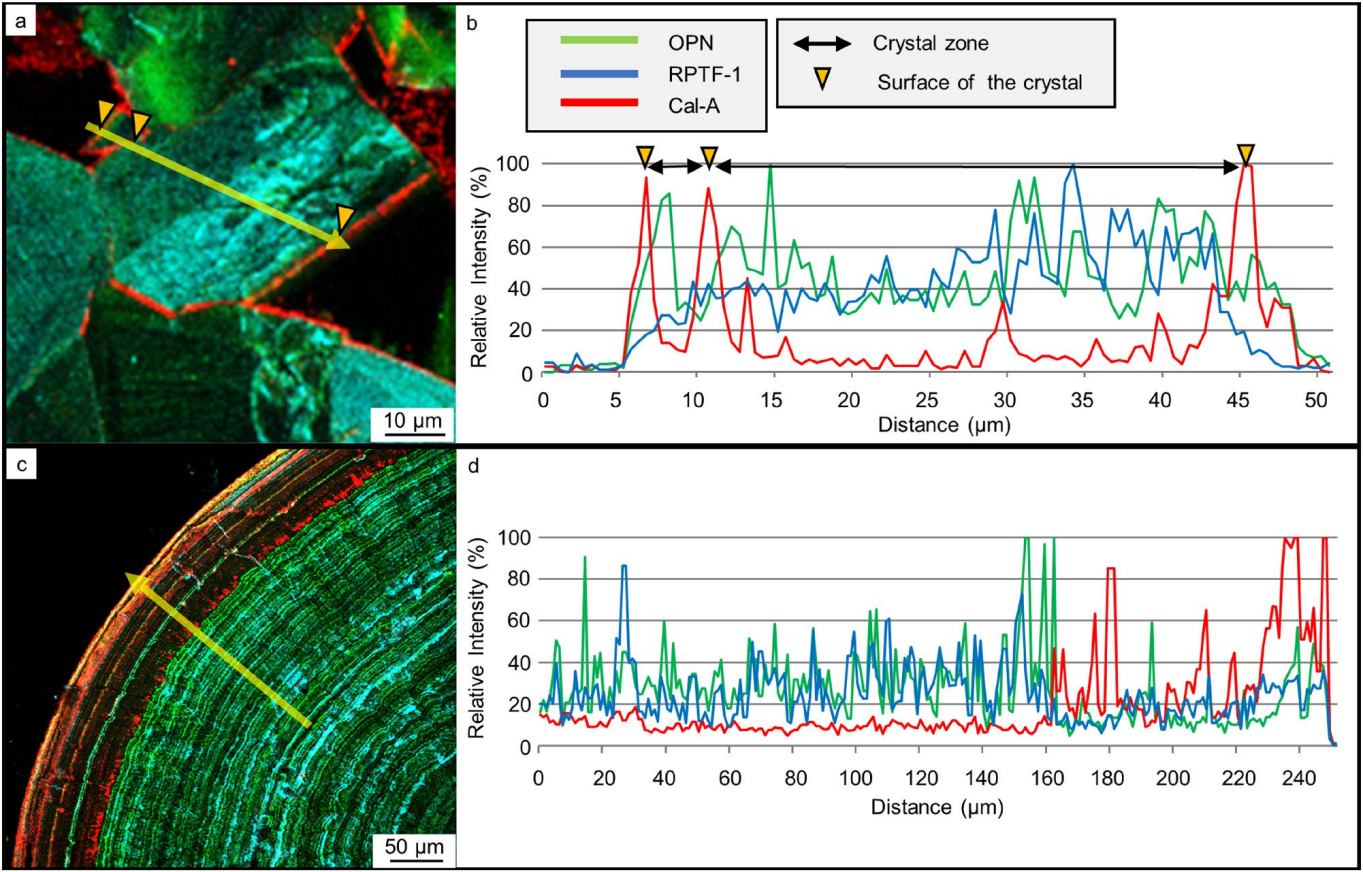
Line intensity profiles of protein IF staining across COM samples. (**a**) Mosaic COM grain with a line profile track shown with a yellow arrow. (**b**) Line intensity profile across the mosaic COM grain in (**a**). (**c**) Conically laminated COM texture with a line profile track shown with a yellow arrow. (**d**) Line intensity profile across the conically laminated COM grain in (**c**).

The mosaic COM texture is the most prevalent texture in CaOx stones (Fig. 1d–f). This texture was observed in 12 samples out of 15 (Table 1). Multi-IF staining of the mosaic COM is shown in Fig. 3a,b. OPN and RPTF-1 were present throughout the COM crystals (Fig. 3c,d). Conversely, Cal-A was exclusively present along the grain boundaries (i.e., on the surfaces of COM grains) (Fig. 3e). We further analyzed a line intensity profile of the proteins, as shown in Fig. 3a, to evaluate each protein distribution pattern in detail (yellow line on Fig. 4a). OPN and RPTF-1 showed higher intensities in the crystal area, whereas Cal-A showed lower intensity in the crystal area (Fig. 4b). The high intensity of Cal-A was observed exclusively along the crystal boundaries. These protein distributions were seen in many samples, although the sizes and shapes of COM grains differed (Supplementary Fig. S3).

**Figure 5:**
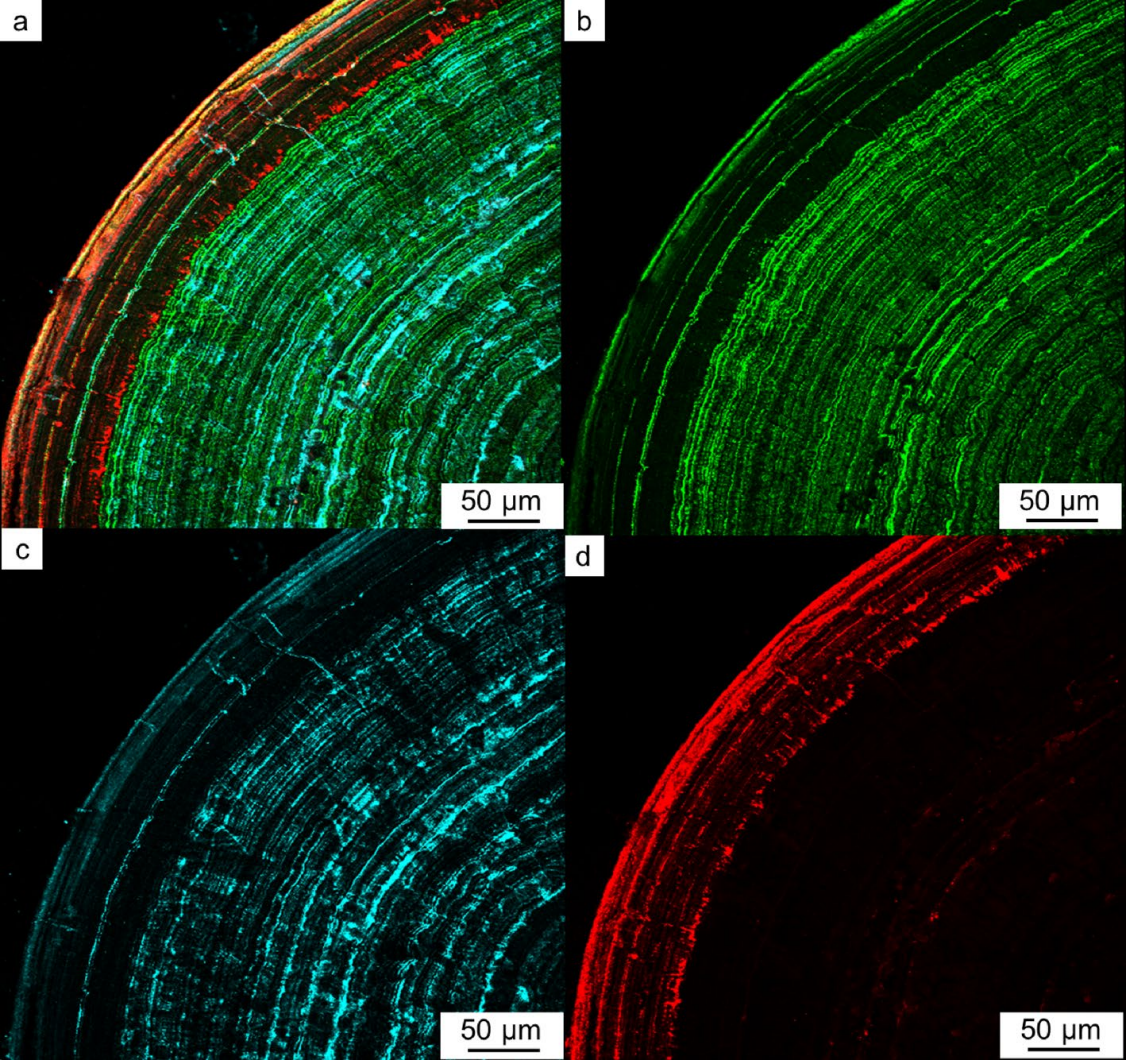
Protein distributions in a conically laminated COM. (**a**) Multi-IF image. (**b**) IF image of OPN. (**c**) IF image of RPTF-1. (**d**) IF image of Cal-A.

**Figure 6:**
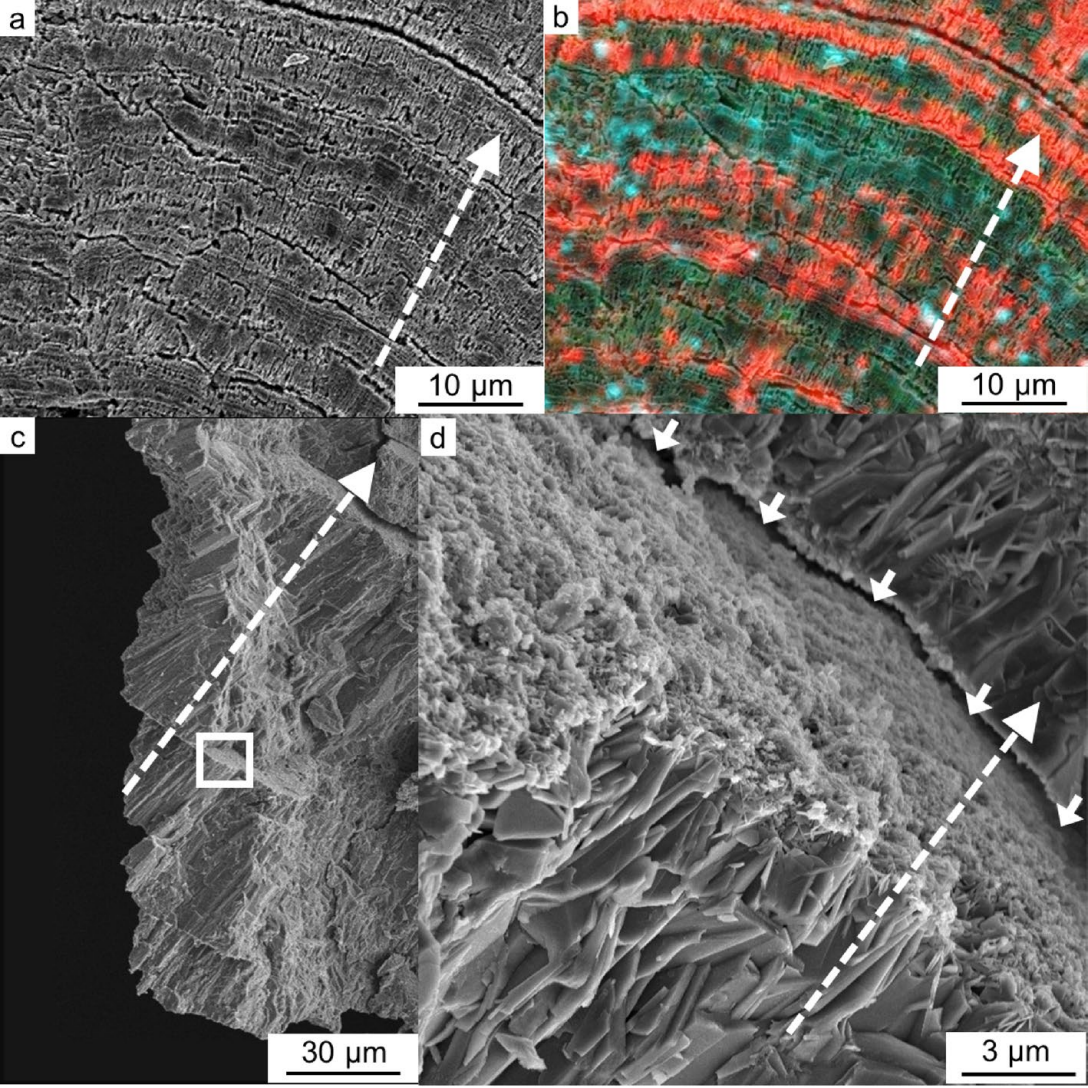
Calgranulin A (Cal-A) distribution in concentric COM. (**a**) Scanning electron microscope (SEM) image of a thin section. (**b**) Merged images of multi-IF staining and SEM of the thin section. The positions of the concentrically aligned gap spaces shown in the SEM image are overlapped with the positions of Cal-A lamination. (**c**) SEM image of a crashed concentric COM. (**d**) Enlarged image of the white box area in (**c**). The directions from the center to the outside of concentric COM stones are shown with dashed white arrows. The gap space between the COM crystals is shown with solid white arrows.

The concentric COM is also a typical texture in CaOx stones (Fig. 1g–i). We found this texture in 10 samples out of 15 (Table 1). OPN, RPTF-1, and Cal-A were distributed in concentric layers (Fig. 5a). OPN and RPTF-1 were present as the constant and regular layers throughout the COM crystals, making a µm-scale interval (Fig. 5b,c). In contrast, Cal-A distribution was composed of irregular and relatively wide-interval layers located in outer surface of the stone (Fig. 5d). SEM observation showed a gap layer close to each Cal-A layer (Fig. 6a,b). Both surfaces creating the gap space were occupied with tiny deposits that were clearly distinct form the major COM crystals composing the concentric COM (Fig. 6c,d). The line intensity profiles of these proteins in the concentric COM showed spikes in each protein profiles (Fig. 4c,d). The profiles of OPN and RPTF-1 were similar (i.e., 5.58 ± 2.70 µm/layer of OPN and 5.99 ± 2.56 µm/layer of RPTF-1) (Supplementary Table S1). However, the Cal-A layers had distinctively large intervals (i.e., 23.17 ± 18.57 µm/layer) than OPN and RPTF-1. Similar distribution patterns and intervals were seen in most of the other 9 samples, although the Cal-A layer was not seen in a few samples (Supplementary Fig. S4 and Table S1).

In this study, the proportions of the three textures in CaOx stones and the distributions of the three proteins in the three textures had no clear relation to age and gender of the stone former, although the amount of the investigated stones was not sufficient for correlation analysis.

**Figure 7:**
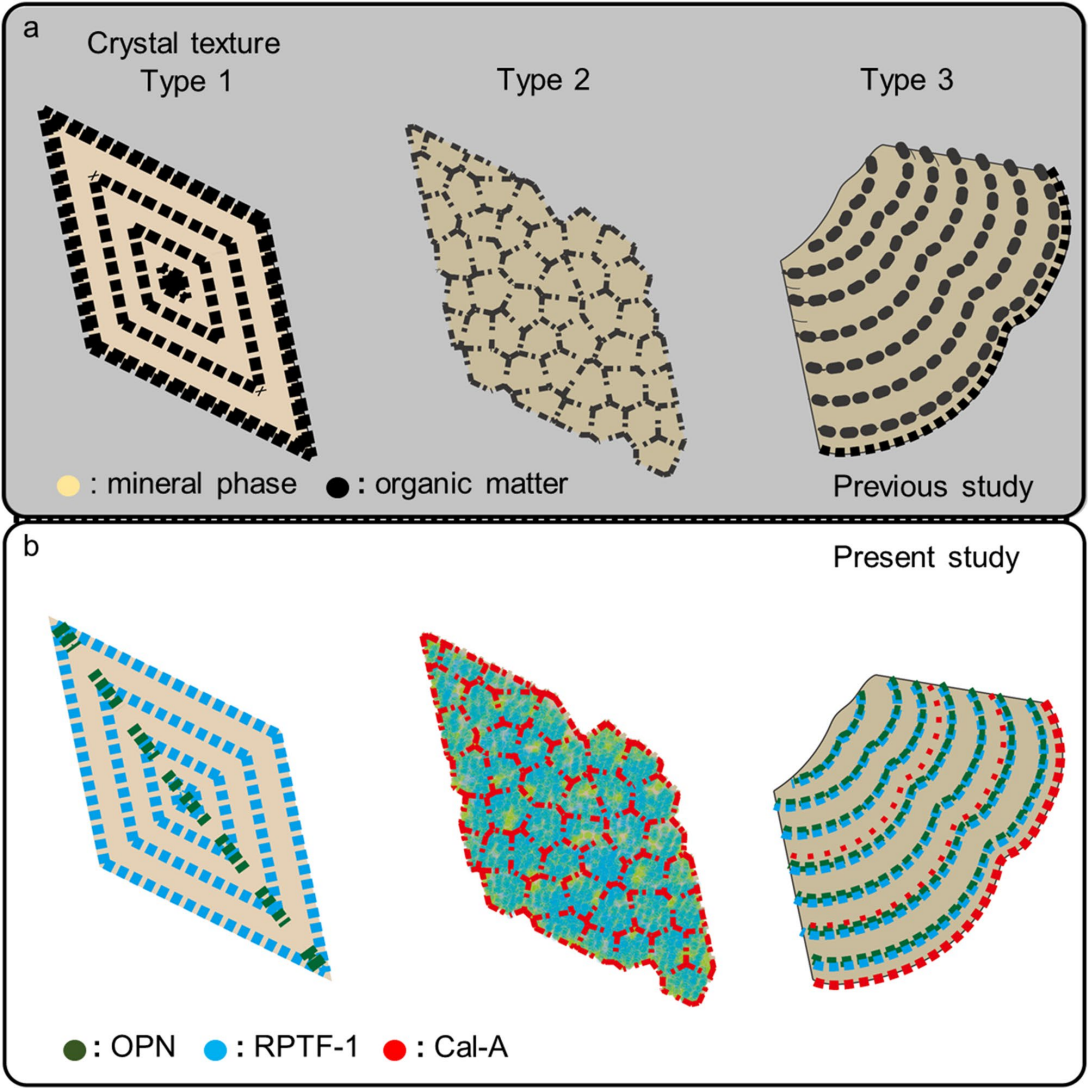
Schematics of the protein distribution in three major texture in CaOx. (**a**) Protein matrix distribution regarded in previous studies (white color: mineral phase black color: organic matter). (**b**) Specific protein distribution found in the present study (green: osteopontin (OPN), blue: renal prothrombin fragment 1 (RPTF-1), and red: calgranulin A (Cal-A). Type1 idiomorphic COD, Type2 mosaic COM, and Type3 concentrically laminated COM.

OPN, RPTF-1, and Cal-A have been known to be present in CaOx stones based on the detection from the extracts of stone powders with electrolysis^9–11^. Micro-scale distributions of OPN in decalcifying CaOx stones with immunocytochemical technique showed OPN distributions in the concentric lamellae in CaOx stones^21,30^. The present method vividly visualized the locations of three different proteins in previously regarded “organic layers” (Fig. 7a,b). The distribution pattern of OPN we show in the present study in the concentric COM is consistent with previous observations. We further found that the distribution pattern of RPTF-1 in the concentric COM is almost the same as those of OPN, whereas the distribution of RPTF-1 in COD crystals is different from those of OPN. Conversely, the distribution pattern of Cal-A is entirely different from the other two proteins. These different micro-scale distributions of OPN, RPTF-1, and Cal-A record the history of CaOx stone formation.

## Discussion

### The primary controlling factor of protein incorporation into crystals

Both OPN and RPTF-1 were present in euhedral COD crystals, mosaic COM grains, and concentric COM (Figs. 2a, 3a and 5a). This finding confirms that these proteins are incorporated in both COD and COM crystals. Cal-A was present in the outside of euhedral COD crystals and around mosaic COM grains (Figs. 2e and 3e). In the concentric COM case, a gap space typically found around the Cal-A layers suggests that the Cal-A layers are not included in the crystals but distributed on the crystals’ surface (Figs. 5d and 6a–d). These distributions indicate that OPN and RPTF-1 tend to be incorporated into CaOx crystals, whereas Cal-A is hardly incorporated.

The protein adsorption and incorporation into CaOx crystals are theoretically influenced by the binding force of their amino acid side chains and other specific properties of respective proteins such as electrostatic negative charge^31,32^. Net charge indicates that OPN and RPTF-1 are more negatively charged than Cal-A, with isoelectric points of OPN, RPTF-1, and Cal-A of 3.5, 2.5–3.0, and 6.5–7.0, respectively^33–35^. The OPN, RPTF-1, and Cal-A are known to have calcium-binding domains^36–38^. These domains may additionally work as local binding sites to the growing crystal surfaces. The calcium-binding domains of OPN, RPTF-1, and Cal-A can bind 10, 7, and 2 calcium ions, respectively^36–38^. Thus, the bulk and local affinities of these proteins to the positively charged Ca on the surface of CaOx would cause the different incorporation efficiency of these proteins into CaOx.

Crystal growth proceeds by incorporating growth units into steps and kink sites that appear on crystal surfaces^39,40^. Three-dimensional compatibilities between proteins and growing crystal surfaces would determine the extent of protein incorporation into the crystal surface. Proteins with a strong binding ability to the growing kink sites and steps tend to be incorporated into the growing crystal more efficiently^41–43^. The presently reported distributions and the calcium affinities of the three proteins suggest that the strong affinity of OPN and RPTF-1 facilitates the binding and incorporation into COD and COM crystals. Conversely, Cal-A, which has a lower affinity, only adheres to the crystal surface but is not incorporated into the crystal. A previous study that showed higher rates of peptide incorporation into COM by more phosphorylated peptides supports this explanation^43^. These discussions would allow us to predict the distributions of many other proteins based on their calcium-binding properties.

### In vivo selective absorption and inhibitory effects of OPN and RPTF-1 on CaOx crystal growth

The euhedral COD crystals that are present predominantly on the periphery of CaOx stone have been regarded to grow with a characteristic laminated texture in which the so-called “organic matter layer” and “mineral layer” alternate each other during crystal growth^44,45^. Although the exact reasons underlying the layer shift remains unclear, potential explanations include the environment changes in the human host, kidney physiology and urine biochemical changes, and kinetic feedback mechanisms that form oscillatory zoning structure found in minerals^8^.

Present results show that OPN and RPTF-1 construct laminated texture corresponding to the {110} and {101} of the COD faces as intracrystalline proteins, respectively, whereas Cal-A is not included in the laminated texture (Fig. 2c–e). This in vivo evidence of surface selective adsorption/incorporation of proteins on/in kidney stone crystals indicates that the distributions of proteins are not identical to the traditional “organic matter layer” observed by optical microscopy (Fig. 7a). In vitro evidence on the selective adsorption of OPN on COD crystal was reported by Chien et al. (2009 and 2018)^46,47^. According to these reports, OPN binds to the typical crystallographic {110} face of COD and incorporates into the mineral phase, being consistent with our results (Fig. 7b). The surface selective adsorption/incorporation has probably resulted from the calcium-binding capacity of each protein and/or different molecular compatibilities between the functional groups on the protein molecules and the arrangement of lattice ions on each crystal surface.

Most proteins reportedly inhibit CaOx crystal growth during stone formation^48,49^. The inhibition of crystal growth by peptides is regarded as through the step pinning and/or the kink blocking^41–43^. In previous in vitro studies, OPN and RPTF-1 are known to be inhibitors in CaOx crystal growth^48,49^. However, the extent of the inhibition in the kidney stone formation remains unclear.

The present observation shows that most kidney stone COD crystals have a typical crystal habit with {101} faces (Fig. 2b). Crystal surfaces that have relatively slow growth rates are generally well developed^40^. This finding indicates that the growth rate of {101} faces of COD is slow compared to other surfaces (e.g., {110}). The preferential adsorption of RPTF-1 that potentially decelerated crystal growth by incorporated in COD was found as a laminated structure along with {101} faces (Fig. 2d). On the other hand, we found {110} faces as OPN laminated structure inside the COD crystal (Fig. 2c). The {110} faces appeared as the OPN laminated structure are more developed than the {110} faces of OPN-free COD crystals obtained in in vitro study^46^. The {110} face of COD with preferential adsorption of OPN is not the crystal face that appears on the COD of the typical crystal habit^47^. Thus, OPN absorption on {110} would extremely decelerate the relative surface growth rate, and the deceleration was slow enough to appear the {110} faces. The emergence of the {110} faces, in other words, changes the general crystal habit in many samples of kidney stones.

COM crystals appear either as a mosaic texture or concentric texture (Figs. 3a and 5a). Both OPN and RPTF-1 are homogeneously present in COM grains in the mosaic texture (Fig. 3c,d). Thus, the effects of these proteins on the growth rate of this type of COM is not clear. In concentric COM, lath-like crystals aligned radially from the center to the outside^50^. The crystal faces of the lath-like crystals are not clear, but the outer surface of the spherical COM that is exposed to urine should have the same crystallographic faces. The present results clearly show that OPN and RPTF-1 have similar distribution line profiles in which they distribute periodically in the concentric COM as approximately 4 to 6 µm-scale interval layers (Figs. 4c,d, 5b,c and Supplementary Table S1). This indicates that OPN and RPTF-1 have a negligible difference in the adsorption and incorporation on the specific crystal faces of COM exposed to urine. Previous in vitro works showed that OPN has inhibitory effects on the COM growth of {100}, {121}, and {010} faces ^51^. When this inhibitory effect is considered, the present result indicates that the concentric COM growth was inhibited by OPN and possibly by RPTF-1 as RPTF-1 has a similar calcium-binding capacity and thus has similar incorporation efficiency to COM.

### In vivo inhibitory effects of Cal-A on CaOx crystal growth

Cal-A adsorption on specific crystal faces of both COM and COD crystals was not seen in the present result (Figs. 2a and 3a). Cal-A has a lower calcium-binding capacity than OPN and RPTF-1 and was present in the outside of euhedral COD crystals and around mosaic COM grains without laminated structure (Figs. 2a,e and 3a,e). However, Cal-A is also known to inhibit CaOx crystal growth in in vitro study^28^. Thus, Cal-A might have worked as a surfactant molecule that affects morphology and crystal growth rate without incorporation into the crystal^39,40,52^. Under regular urine conditions (i.e., without irregular high Cal-A concentration), Cal-A may have a lower inhibitory effect than OPN and RPTF-1 on the crystal growth of COM and COD since OPN and RPTF-1 inhibit the development of steps on crystal faces through pinning and/or blocking by incorporation in crystals. Therefore, the calcium-binding capacity of proteins in kidney stones would help evaluate the extent of their inhibitory effects on CaOx growth.

In concentric COM, Cal-A showed non-periodical layers that had distances from 20 to 50 µm (Figs. 4c,d, 5d and Supplementary Table S1). Cal-A has been identified in stone formers’ urine^28^. Thus, fluctuations in its concentration in urine should naturally occur. However, due to its lower affinity to calcium ion, small fluctuations in Cal-A concentration might not be recorded in the COM texture. Cal-A is excreted in urine non-periodically as anti-inflammatory proteins in response to inflammation^53,54^. Thus, the non-periodical layers of Cal-A may record occasional high concentrations of Cal-A in urine due to irregular environmental changes, such as infection, injury, and bleeding. Its inhibitory effect on COM growth would strongly depend on its concentration around growing crystals as several peptides have such dependency on the calcite crystal growth^42^. Cal-A may weakly decelerate COM growth by partly occupying the growing surface when its concentration around the crystal is low because the protein is not prone to be incorporated in CaOx. In concentric COM, gap layers surrounded by tiny deposits found by SEM observation present almost identical place as the Cal-A layers found by multi-IF imaging (Fig. 6a-d). Therefore, when the Cal-A layer becomes thick due to its irregularly high concentration by some biological changes, Cal-A may work as a potent inhibitor of CaOx growth by widely occupying the growing surface. Inflammation, which is a trigger of Cal-A secretion, may also affect a different step of the stone formation because a type of white blood cells (i.e., macrophages) that have promotion and inhibitory effects on kidney stone formation have been found in experiments using a kidney stone model mouse^16^.

### The possible application of the multi-IF imaging to overall kidney stone formation

The present work expanded the application of multi-IF imaging of proteins to harder biomineral samples, i.e., kidney stones. Crystal nucleation, crystal growth, crystal aggregation, and crystal adhesion have been regarded as important steps in kidney stone formation. OPN, RPTF-1 and Cal-A are reported as inhibitors of nucleation, growth, and aggregation of CaOx by in vitro studies^48,49^. The present expanded application of multi-IF imaging provides in vivo evidence of the inhibitory effect of CaOx growth by proteins that was implied by the previous in vitro studies.

Investigations on the effects of proteins in each step have been conducted by in vivo studies in mice as well as numbers of in vitro studies^14,15,26^. For example, a critical renoprotective role of OPN as an inhibitor of crystal formation and an inhibitor of crystal adhesion has been reported based on in vivo experiments in mice^26^ whereas, the promotion of crystal adhesion to tubular epithelial cells by OPN and the enhancement of CaOx stone formation have been reported based on more recent in vivo experiments using OPN-knockout mice^14,15^. A completely different crystal morphology found in kidney stones of an OPN-knockout mouse also supports the effects of OPN in multiple steps of kidney stone formation. The present visualization method would be useful in evaluating protein effects in these in vitro studies in mice and even useful in evaluating different steps of human kidney stone formation. This novel analysis would allow us to interpret the in vivo effects of different proteins on CaOx stone formation that can open up a route for the pathological examination and personalized medicine to manage the human kidney stone disease.

## Methods

### Ethics statement

The research project presented in this paper was approved by the institutional review board of the graduate school of medicine, Nagoya City university. All methods were carried out in accordance with the relevant guidelines and regulations. Written informed consent from all subjects was obtained according to procedures approved by the ethical committee board.

### Stone collection and preparation of stone sections

Kidney stones were collected from patients and were analyzed at the Nagoya city university in Japan. Fifteen CaOx kidney stone samples were selected from our thousands of human kidney stone collections based on the information of bulk infrared spectroscopy (IR) and thin sections were prepared. The bulk mineralogical composition of the stone was estimated with the IR analysis. A petrological thin sectioning methodology, originally designed for geological investigations, was applied to this kidney stone investigation^55^. Kidney stone samples were entirely embedded in epoxy resin and cut. The cross-section was ground with abrasives (SiC and Al_2_O_3_), and then the polished face was adhered to a glass slide using epoxy resin. A second cut was performed to make a 1-mm thick sample parallel to the glass slide. The specimen was then polished down to a thickness of 20–30 µm and finished with the polish by diamond slurry.

### Polarized microscopy and Fourier transform infrared spectrophotometry

The optical features of each crystal composing the kidney stones were observed by polarized microscopy with the 20–30 µm thick stone fragment sections. The surface index of each COD plane was determined by comparing the observation with the typical COD crystal shape calculated by VESTA^56^. Based on the optical features, crystals were classified, then analyzed with a Fourier transform infrared spectrophotometer (FT/IR6100, JASCO). The measurement wavenumber range was 7800–350 cm^-1^. All measurements were conducted at ambient temperature. The measurement spot size was set at 20 × 20 µm^2^. Crystal phases were identified based on the obtained IR spectrum with the RRUFF database.

### Multicolor immunofluorescent staining of the protein matrix

The stone sections used for the analysis of mineral phases were also used for the analysis of protein distributions with Multi-IF staining. The thin sections were treated with a citrate solution (pH 6.0) for 1 min for a minimum etching. The section was washed with phosphate-buffered saline (PBS), then blocked for 60 min in 1% Bovine Serum Albumin in phosphate-buffered saline with Tween 20 (PBST). After the blocking, the section was incubated with primary antibodies overnight at ambient temperature. The primary antibodies used were mouse monoclonal anti-calgranulin A (1:100 dilution, Santa Cruz Biotechnology, sc-48352), rabbit polyclonal anti-osteopontin (1:100 dilution, Santa Cruz Biotechnology, sc-20631), and sheep monoclonal anti-human prothrombin Fragment 1 (1:500 dilution, Cedarlane Ontario, Canada, CL20111AP). The section was then washed 3 times with PBS for 10 min before incubation with fluorescently conjugated secondary antibodies for 1 h, protected from light. The fluorescently conjugated secondary antibodies used were anti-Rabbit IgG (H + L) Cross-Adsorbed conjugated to Alexa Fluor 488, anti-Mouse IgG (H + L) Cross-Adsorbed conjugated to Alexa Fluor 546, and anti-Sheep IgG (H + L) Cross-Adsorbed conjugated to Alexa Fluor 647. After extensive 3 times washing with PBS for 10 min, fluorescence was detected using a confocal Auto-Fluorescence Microscopy (Nikon A1R). Excitation and emission wavelengths collected included 487 nm excitation (emission collected between 500 and 550 nm), 561 nm excitation (emission collected between 570 and 620 nm), and 639 nm (emission collected between 663 and 738 nm) for OPN, RPTF-1, and Cal-A, respectively.

Auto-fluorescence (AF) was observed from a part of the samples, but the signal intensity was far lower than the protein signals discussed in this study. The binding of antibodies that are not specific to the target proteins was also evaluated. The absence of IF signals from the non-specific antibody was confirmed. Negative control tests were performed to evaluate the absence of false-positive signals (Supplemental Fig. S5). For antibody staining, isotype controls were used to detect any non-specific binding. Specifically, primary antibodies used for controls were rabbit (DA1E) mAb IgG XP isotype control (1:100 dilution Cell Signaling), mouse (G3A1) mAb IgG1 isotype control (1:100 dilution Cell Signaling), and sheep mAb IgG isotype (1:100 dilution Novus) using the same fluorescently labeled secondary antibodies as above. The line profiles were constructed with ImageJ, showing the highest and lowest contrast point as 100% and 0%, respectively, for each protein.

## Supplementary Information

Supplementary Information 1.
