# Supplementary material for: Multicolor imaging of calcium-binding proteins in human kidney stones for elucidating the effects of proteins on crystal growth
Source: Sci Rep. 2021 Aug 26;11:16841. doi: 10.1038/s41598-021-95782-1 (PMC8390759; doi:10.1038/s41598-021-95782-1)
Supplement: Supplementary file 1 — Supplementary Information 1. [file 41598_2021_95782_MOESM1_ESM.docx]

**Supplementary Information**

**Manuscript: Multicolor imaging of calcium-binding proteins in human kidney stones for elucidating the effects of proteins on crystal growth**

**Yutaro Tanaka, Mihoko Maruyama*, Atsushi Okada*, Yoshihiro Furukawa, Koichi Momma, Yuki Sugiura, Rie Tajiri, Koichi P. Sawada, Shunichi Tanaka, Kazufumi Takano, Kazumi Taguchi, Shuzo Hamamoto, Ryosuke Ando, Katsuo Tsukamoto, Masashi Yoshimura, Yusuke Mori, Takahiro Yasui.**

**This PDF file includes:**

**Supplemental Fig. S1**. Identification of the COD crystal faces.

**Supplemental Fig. S2.** Protein distributions in a bipyramid COD texture.

**Supplemental Fig. S3**. Protein distributions in mosaic COM texture.

**Supplemental Fig. S4.** Protein distributions in concentrically laminated COM texture.

**Supplemental Fig. S5.** Microscope images of negative control of the Multi-IF staining:

**Supplemental Table S1.** Summary of the interval protein layers of concentrically laminated COM crystals.


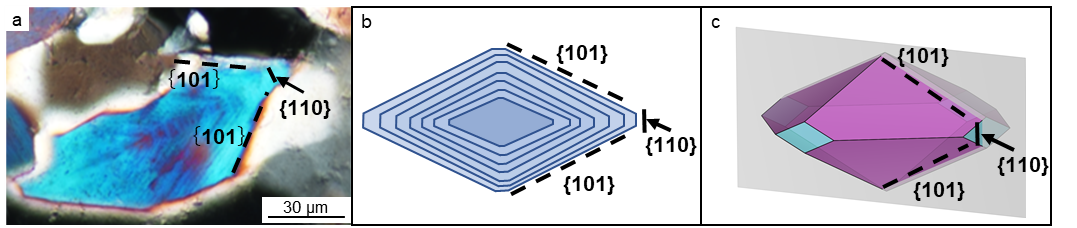


Supplementary Fig. S1. Identification of the COD crystal faces. (a) Thin section of COD under polarized microscopy. (b) Schematic of the cross-sectional view of COD. (c) Crystallography of typical COD in kidney stones visualized by VESTA.


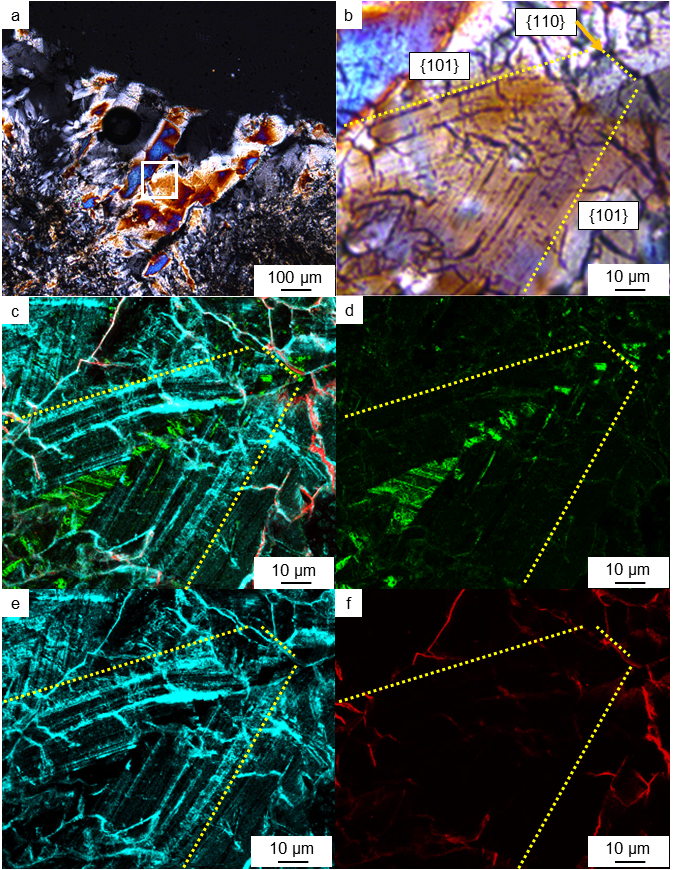


Supplementary Fig. S2. Protein distributions in a bipyramid COD texture. (a) Optical image. (b) Enlarged image of the white box area in (a). (c) Multi-IF image. (d) IF image of OPN. (e) IF image of RPTF-1. (f) IF image of Cal-A. Crystal faces are shown with yellow dotted lines and those indexes.


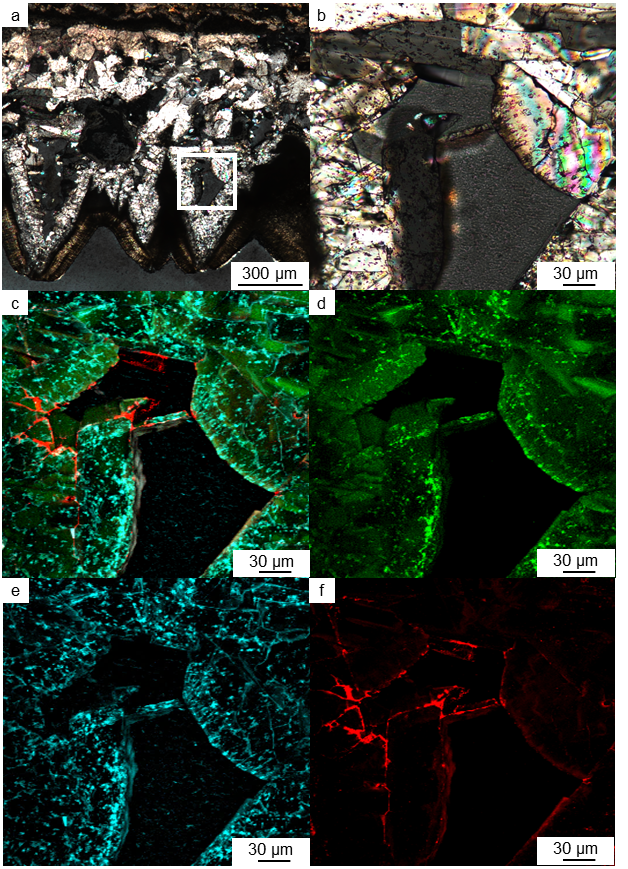


Supplementary Fig. S3. Protein distributions in mosaic COM texture. (a) Optical image. (b) Enlarged image of the white box area in (a). (c) Multi-IF image. (d) IF image of OPN. (e) IF image of RPTF-1. (f) IF image of Cal-A. Crystal faces are shown with yellow dotted lines.


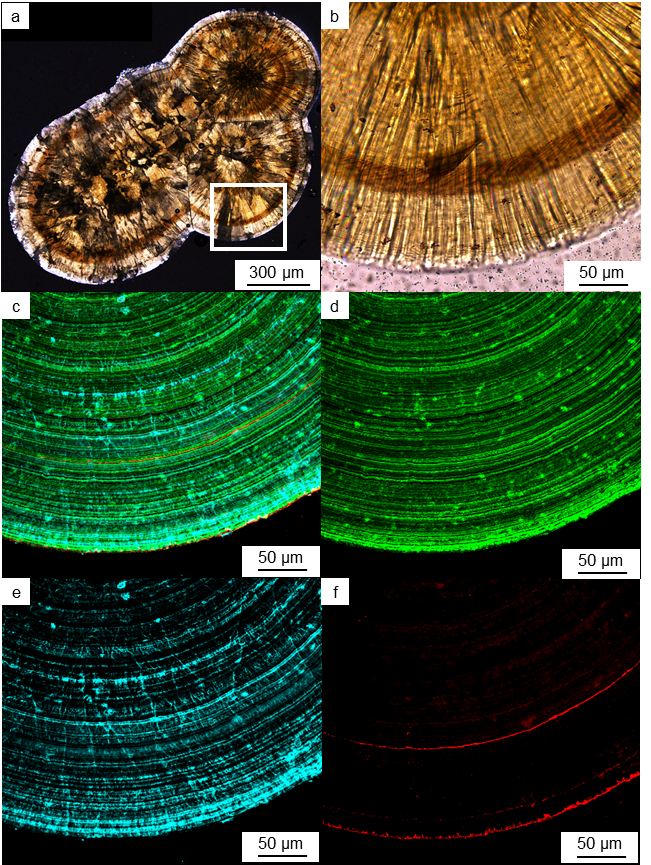


Supplementary Fig. S4. Protein distributions in concentrically laminated COM texture. (a) Optical image. (b) Enlarged image of the white box area in (a). (c) Multi-IF image. (d) IF image of OPN. (e) IF image of RPTF-1. (f) IF image of Cal-A.


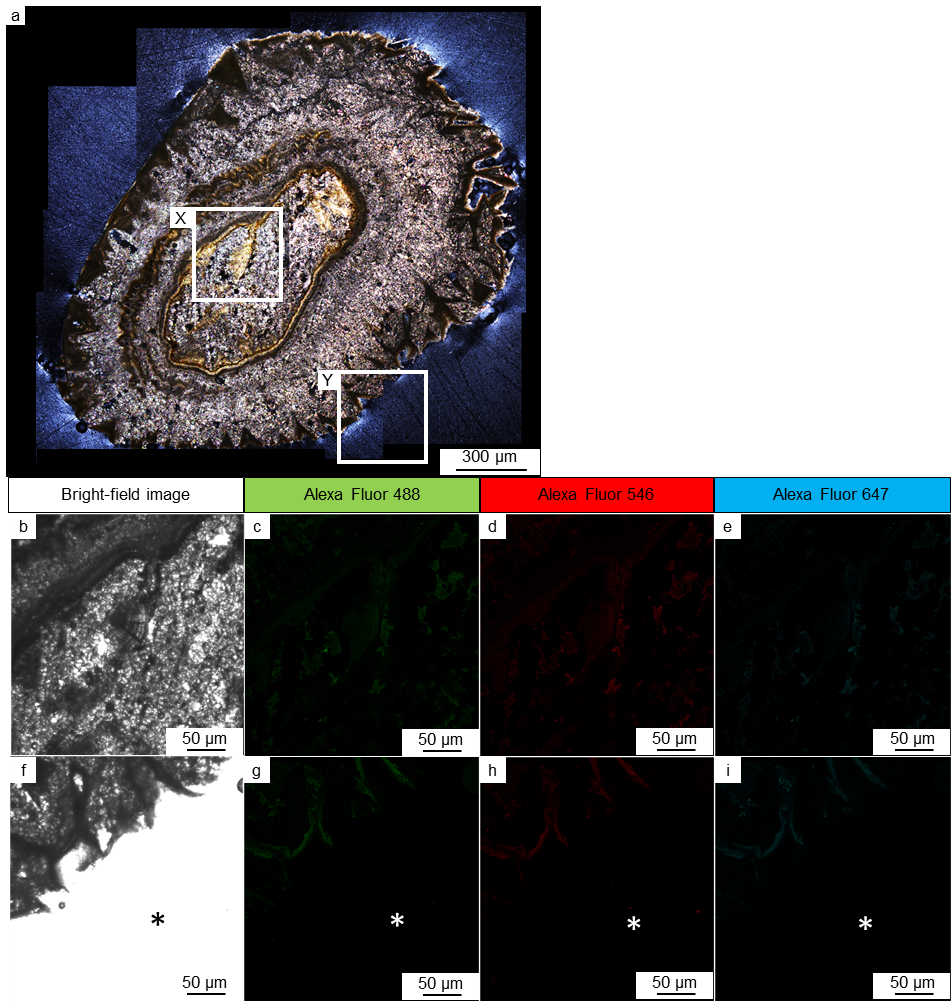


Supplementary Fig. S5. Microscope images of negative control of the Multi-IF staining: (a) Stone section under polarized microscopy. The region X is occupied with kidney stone sample, whereas the region Y is dominated by the epoxy resin used to fix the stone. (b) Optical image of region X. (c) IF staining image of Alexa Fluor 488 of region X. (d) IF staining image of Alexa Fluor 546 of region X. (e) IF staining image of Alexa Fluor 647 of region X. (f) Optical image of region Y. The epoxy resin region is marked with an asterisk (g) IF staining image of Alexa Fluor 488 of region X. (h) IF staining of Alexa Fluor 546 of region Y. (i) IF staining image of Alexa Fluor 647 of region X.

Supplementary Table S1. Summary of the interval protein layers of concentrically laminated COM crystals.


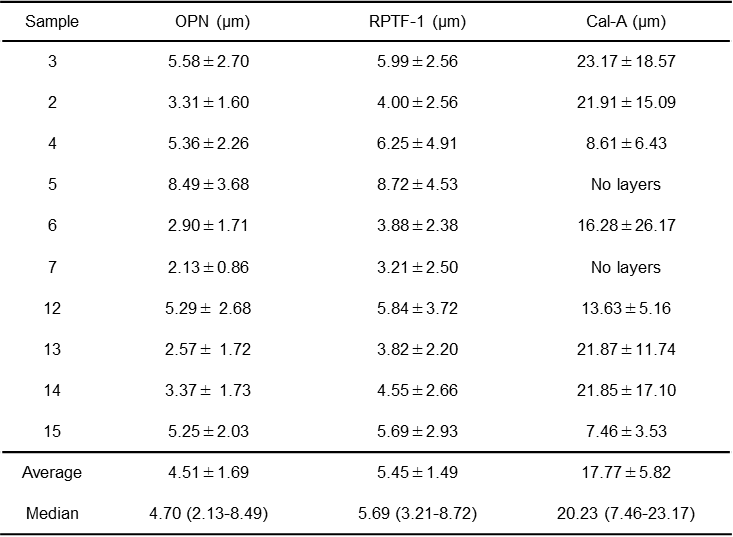


OPN: osteopontin, RPTF-1: renal prothrombin fragment-1, and Cal-A: calgranulin-A.
